# Propagation, not aggregation: linking individual psychological flexibility to organizational functioning

**DOI:** 10.3389/fpsyg.2026.1918803

**Published:** 2026-09-17

**Authors:** Simone Guida, Claudia Minutola, Giovambattista Presti

**Affiliations:** 1 Department of Business, Law, Economics and Consumer Behavior “Carlo A. Ricciardi”, Università IULM, Milan, Italy; 2 Behavior and Brain Lab IULM – Neuromarketing Research Center, Università IULM, Milan, Italy; 3 Department of Human and Social Sciences, University of Enna “Kore”, Enna, Italy

**Keywords:** acceptance and commitment therapy (act), occupational health, organizational flexibility, prosocial, psychological flexibility

## Abstract

Individual psychological flexibility is reliably associated with worker health, and interventions that raise it improve how individuals fare; yet such interventions do not reliably change the organizations those individuals belong to. This paper takes that disparity as its starting point and argues that the missing link is propagation rather than production: organizational flexibility is not the aggregate of flexible individuals but a pattern of coordinated, values-consistent action that is retained and spread across an organization, or absorbed and extinguished, depending on the conditions it meets. Integrating Acceptance and Commitment Therapy, the Prosocial approach, and a multilevel account of selection by consequences, the paper proposes a three-level model in which whether an increase in individual flexibility reaches the organizational level depends on two conditions: the leverage of the affected members over the consequences their colleagues encounter, and whether the surrounding culture reinforces or penalizes values-consistent action. Bond’s emphasis on the flexibility of leaders is recovered as a high-leverage special case of this more general mechanism. The model is developed conceptually and yields a set of testable propositions, together with the measures their testing would require. Throughout, worker health is treated as a value worth pursuing in its own right rather than as an instrument of performance.

## 1 Introduction

The literature on financialization shows that, since the 1970s, the growing weight of financial markets, institutions and metrics has progressively reoriented capitalist accumulation and corporate governance, turning finance not merely into a sector but into a broader logic for coordinating organizational decision-making (Epstein, 2005; Krippner, 2005; van der Zwan, 2014). In this context, the rise of shareholder value has shifted many large corporations away from a logic of retain and reinvest, oriented toward long-term growth, innovation and the development of productive capabilities, toward a logic of downsize and distribute, in which dividends, share buybacks, quarterly results and market valuation become central criteria of performance (Lazonick and O’Sullivan, 2000). Empirical evidence suggests that this reallocation of priorities can reduce real investment and productive accumulation, as resources are increasingly channeled toward financial profits, shareholder payouts and short-term earnings management (Stockhammer, 2004; Orhangazi, 2008; Graham et al., 2005; Mertens, 2025). This produces a tension between value creation and value extraction: while innovation depends on cumulative, collective and uncertain processes, financial capitalism tends to concentrate returns in the hands of actors able to appropriate financially the value generated by organizations, workers and public investment (Lazonick and Mazzucato, 2013).

This tension between value creation and value extraction does not stay on the balance sheet; it reaches into the conditions under which people work. Where short-term financial metrics dominate the criteria of organizational performance, the consequences that shape everyday behavior are increasingly tuned to reward extraction and to penalize the slower, values-consistent action on which both worker health and organizational adaptability depend. We take worker health to matter in its own right, and not only for its effects on performance, and it is from this standpoint that the present paper asks what allows healthy, values-consistent action to take hold across an organization, or prevents it.

Systematic reviews indicate that psychosocial risk factors, in particular the combination of high job demands and insufficient resources, predict adverse health outcomes such as burnout and disengagement across sectors and occupational groups (Galanakis and Tsitouri, 2022; Bukhtiyarov et al., 2021; Lulli et al., 2021). The Job Demands-Resources model (JD-R; Bakker and Demerouti, 2016) has been the dominant framework in this field, identifying the imbalance between demands and resources as the core link between organizational conditions and individual health. As an account of which conditions covary with health it is well supported. What it does not do is specify the contingencies through which those conditions come to select, or fail to select, coordinated patterns of healthy, values-consistent action; it describes the conditions under which workers become unwell without specifying what would produce health at the level of the organization as a whole.

One response has been to intervene directly on the individual. Psychological flexibility (PF), the capacity to stay in contact with the present and act in the service of chosen values even in the presence of difficult thoughts and feelings, is robustly associated with occupational health outcomes (Roche et al., 2026; Prudenzi et al., 2021), and ACT-based interventions reliably improve it. Yet there is now direct evidence that individual-focused interventions, on their own, do not carry through to the organization. In the largest study of its kind, covering 46,336 workers across 233 UK organizations, participants in individual-level wellbeing interventions including mindfulness, resilience training, and wellbeing apps were no better off than non-participants across a range of wellbeing outcomes, with some indications of harm (Fleming, 2024). Individual ACT interventions are one instance of this broader class. The point is not that raising individual flexibility lacks value, but that whether a change produced in some individuals carries through to the organization is a separate question, and one the evidence leaves almost entirely open: the superiority often assumed for organizational-level interventions is itself not firmly established, and the mechanism by which an individual change might propagate across an organization has rarely been measured at all.

This is the gap the present paper addresses. Interventions that raise individual psychological flexibility improve individual health, but they translate into organizational change only when values-consistent action reaches people positioned to alter the consequences their colleagues encounter, and unfolds within a culture that reinforces rather than penalizes that action. The paper specifies these two conditions of propagation, which neither the Job Demands-Resources tradition nor Bond’s organizational flexibility model makes explicit, and frames them as testable propositions.

To address this gap, the present paper proposes a three-level model that treats the propagation of values-consistent action, rather than its production in individuals, as the central problem. The most developed prior attempt to extend the flexibility logic to the organizational level is Bond's (2018) organizational flexibility model, which holds that organizations, like individuals, depend on flexibility to sustain both performance and health, and specifies organizational characteristics functionally analogous to the processes that constitute flexibility in a person. Bond identifies the flexibility of leaders as one route by which these characteristics can be put in place, a top-down route from those who shape an organization to the organization itself; what the model leaves open is the more general mechanism by which an increase in flexibility among members comes to be retained and propagated rather than absorbed and extinguished.

The model proposed here is organized around three levels. At the first is the individual: psychological flexibility as a process governing how a worker relates to their own experience, values, and behavior in the workplace. At the second is the organization: the conditions, including shared purpose, commons-based coordination, and norms that support cooperation, which reinforce or undermine values-consistent action across the workforce. The third is not a further tier in a hierarchy but the level at which organizational flexibility itself is located: a recurring pattern of coordinated, values-consistent action that is retained and propagated when the wider context selects for it, and that decays when it does not.

Drawing on a multilevel selection logic, we argue that what determines the fate of an increase in individual flexibility is whether it falls on members positioned to shape the consequences their colleagues encounter, and whether the surrounding culture reinforces rather than penalizes action taken in the service of shared values. Where these conditions hold, values-consistent action is selected, retained, and amplified across the organization; where they do not, it is absorbed or extinguished, however flexible the individuals involved. Alignment between individual flexibility and a values-selecting culture is therefore necessary but not sufficient on its own: what does the work is the selective context into which flexibility is introduced.

Each level is grounded in an established framework, Acceptance and Commitment Therapy at the individual level and the Prosocial model (Atkins et al., 2019) at the collective level, and each is connected to occupational health predictions that distinguish genuine value alignment from mere formal compliance and from value conflict. We develop the model conceptually and derive from it a set of testable propositions; the empirical program it implies, including the measures it would require, is set out in the final section.

## 2 Theoretical background

### 2.1 Psychological flexibility and ACT

The model of psychological flexibility on which this paper draws comes from Acceptance and Commitment Therapy, and equally Acceptance and Commitment Training in its non-clinical uses (ACT; Hayes et al., 1999). Although ACT originated in clinical work, the flexibility model it rests on is not a model of psychopathology. It is a general account of human functioning: a description of how people either act in ways that adjust to changing conditions in the service of what matters to them, or fall into rigid patterns that continue regardless of whether they work. This is why the model travels beyond the clinic. A substantial share of its applications lie in non-clinical settings concerned with functioning and wellbeing, including workplaces, schools, and sport. Both ACT and the present paper rest on functional contextualism, a philosophy of science whose aim is to predict and influence the actions of whole organisms in context, individually and in groups (Hayes, 1993; Biglan and Hayes, 1996; Hayes et al., 2012). Because behavior is always an act-in-context, the framework lends itself to extension beyond the individual.

Psychological flexibility (PF) is the core of this model. It is best understood not as something a person has but as a pattern of action: staying in contact with the present and persisting in or changing behavior in the service of chosen values (Hayes et al., 2006). Read in functional terms, flexibility is not change for its own sake, nor simply moving with whatever a situation demands. What is selected is action that serves chosen values, so that persisting and changing are both flexible when the values direction calls for them, while rigidity is action that continues, or shifts, without regard to whether it serves that direction. PF is therefore not a personality trait but a repertoire of interrelated behavioral skills, organized around six processes (acceptance, cognitive defusion, present-moment awareness, self-as-context, values clarification, and committed action) commonly depicted as the hexaflex (Figure 1). These processes can be directly targeted through intervention, which distinguishes PF from constructs that only predict outcomes and gives the framework a practical advantage for organizational health research.

**Figure 1:**
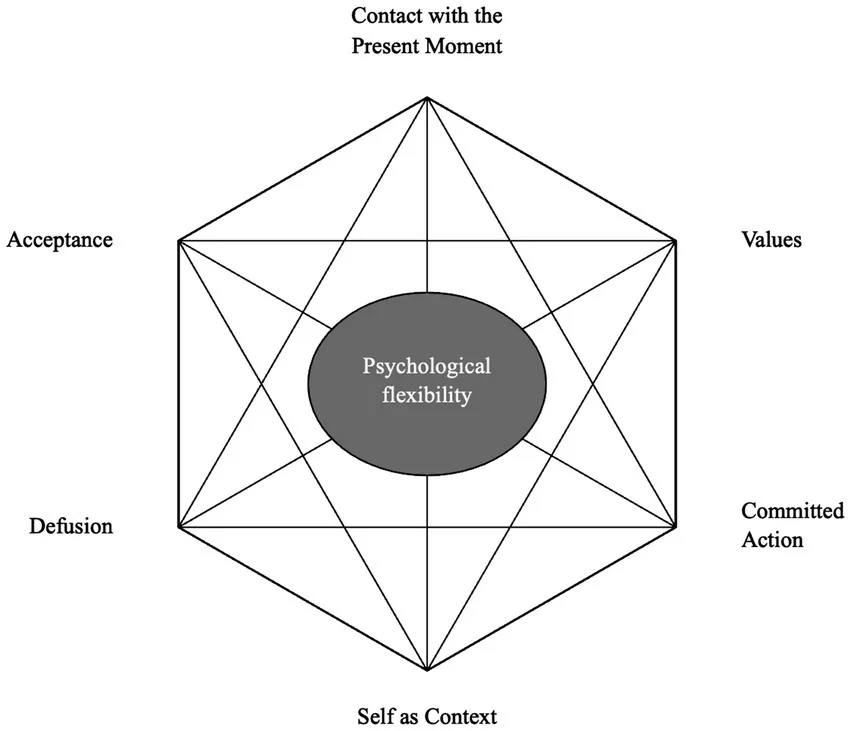
ACT’s psychological flexibility model. Conceptual model from Hayes et al. (2006).

A point of particular relevance is that fusion and defusion are not fixed features of a person but positions on a continuum set by context. Any individual, and as this paper will argue any organization, can move along it depending on the conditions to which it is exposed. A culture that rewards emotional suppression or penalizes values-based dissent is precisely such a condition, one that can shift both individuals and groups toward the rigid end regardless of any individual-level intervention.

Empirical support for ACT in occupational settings is substantial. Systematic reviews and meta-analyses report consistent reductions in burnout, work-related stress, and exhaustion, with psychological flexibility identified as the primary mechanism of change. A large-scale study across three Scandinavian countries found that organization-wide ACT-informed training improved stress resilience and reduced exhaustion, with effects attributed to changes in acceptance and defusion. Mediation analyses confirm that ACT works through changes in psychological flexibility rather than merely alongside them, establishing not only that it works but why. It is worth noting, however, that even when such training is delivered across an entire organization, the outcomes measured are individual ones; whether it changes the organization as a whole has not been established, and it is here that the gap this paper addresses begins.

Notwithstanding this evidence, individual-level ACT interventions carry an inherent limitation in organizational contexts. Psychological flexibility is not context-independent: it is continuously shaped by relational, group-level, and structural conditions. This is what motivates extending the model to the collective and organizational levels addressed in the sections that follow.

### 2.2 From the individual to the collective: prosocial

The transition from individual psychological flexibility to organizational functioning is the central theoretical challenge of the present model, and it is the transition the Prosocial framework (Atkins et al., 2019) is designed to address. Prosocial integrates ACT-based psychological flexibility with multilevel selection theory and Ostrom’s (1990) core design principles for effective group functioning, giving a grounded account of how individual psychological processes can be deliberately cultivated to support cooperative behavior at the collective level (Figure 2). The generality of these principles rests on an evolutionary foundation: Wilson et al. (2013) showed that the principles Ostrom derived for common-pool resource groups follow from the basic dynamics of cooperation and apply to virtually any group whose members must coordinate to achieve shared ends, not only those managing natural resources.

**Figure 2:**
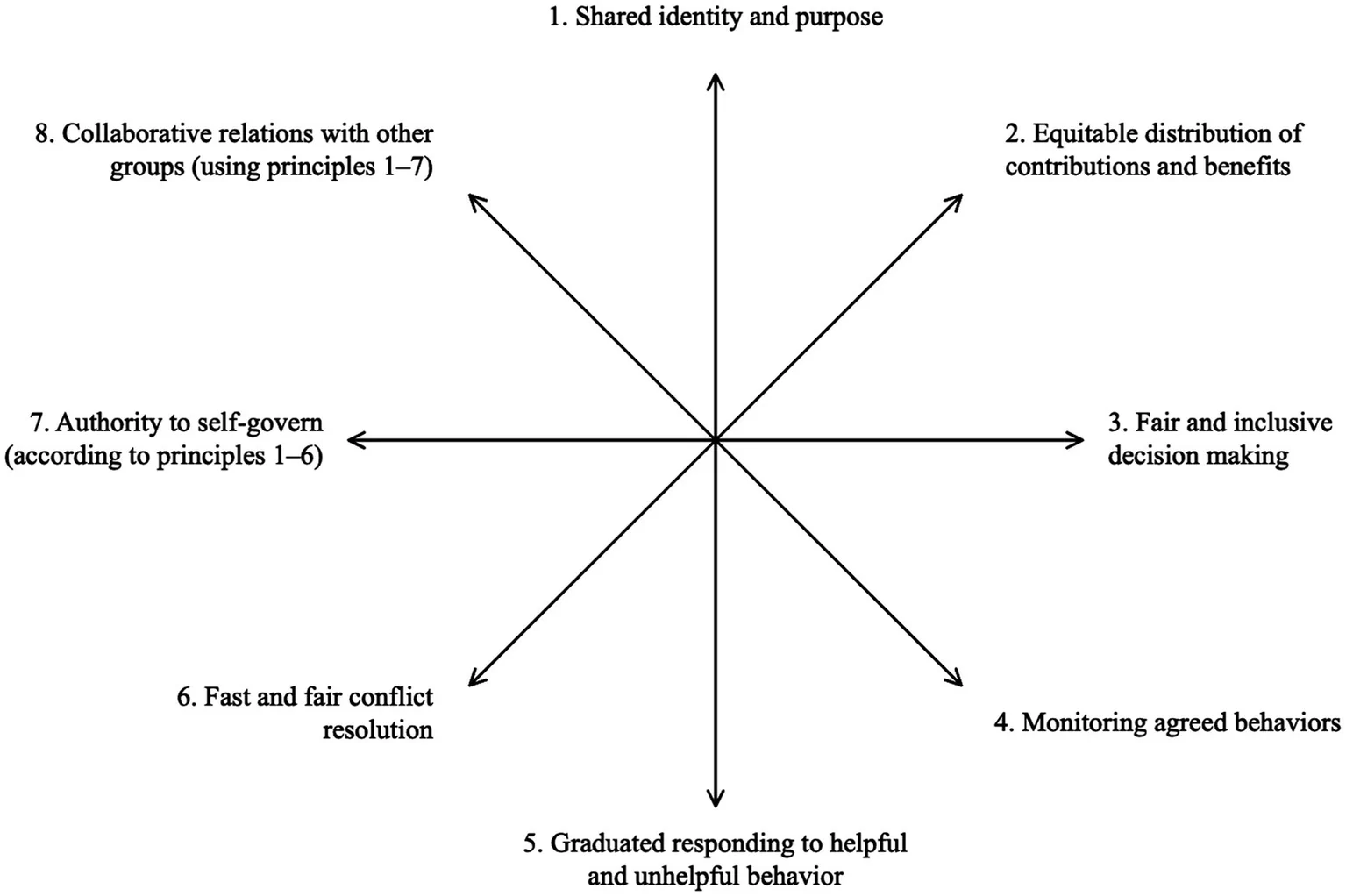
Prosocial core design principles. Conceptual model from Atkins et al. (2019)/Ostrom’s core design principles.

A foundational premise of the approach is that psychological flexibility, the capacity to act in accordance with chosen values even in the presence of difficult thoughts and feelings, is not solely an individual matter. Atkins et al. (2019) argue that the same processes that constitute psychological flexibility in a person (values clarification, cognitive and emotional flexibility, committed action, perspective taking, and present-moment awareness) can be exercised at the level of a group, supporting coordinated, values-consistent action across its members. The claim that this scaling is more than metaphor rests on a concrete method: the ACT matrix (Polk, 2014), a tool for mapping the toward and away dimensions of experience, can be used at the individual, group, and inter-group levels, helping members build a shared sense of purpose and reduce defensive, avoidance-driven behavior within and between groups.

Central to the framework is a distinction that Atkins et al. (2019) place within a multilevel selection perspective: between action reinforced by its immediate consequences for the individual and action that serves the longer-term outcomes a group depends on. The two can pull apart. Behavior that pays off for one member in the short term can undermine outcomes that only the coordinated action of many members produces. This is the tension the multilevel selection tradition illustrates with cancerous cells whose local proliferation comes at the cost of the organism. Perspective taking works on this tension by bringing the longer-term and shared consequences of action into contact with the individual’s behavior, alongside its immediate private payoff. In this sense psychological flexibility does not only serve the individual: it makes prosocial action possible, by allowing short-term avoidance to be subordinated to longer-term, values-consistent commitments held in common.

Of particular relevance here is what happens to values when they are shared. Atkins et al. (2019) argue that as values come to be held in common, they change what reinforces the members of a group, so that coordinated action consistent with those values is increasingly selected and sustained, not only the action of single individuals. It is this process, by which individually held values are extended into a common purpose that members act on together rather than merely endorse, that links individual psychological flexibility to organizational culture. A group whose members are psychologically flexible is more likely to develop the trust, the longer-term orientation, and the concern for shared outcomes that Atkins et al. (2019) identify as conditions for effective cooperation.

Empirical support is provided by Wilson et al. (2020), who surveyed 490 participants rating both a work group and a non-work group on core design principle implementation and on five group outcomes. Better implementation was positively associated with commitment, cooperation, psychological needs satisfaction, group satisfaction, and trust across both group types. Work groups scored lower than non-work groups on all eight principles, with the largest deficits on local autonomy (CDP7), shared identity and purpose (CDP1), and inclusive decision-making (CDP3), precisely the principles most directly tied to the values-based functioning the framework is designed to build.

A culture that suppresses psychological flexibility, by rewarding avoidance, penalizing openness, or subordinating purpose to short-term gain, works in the opposite direction, undermining the conditions under which prosocial action can be sustained. Members and the culture they act within shape each other over time: action consistent with shared values changes the conditions under which others act, and those changed conditions support further such action, or, where the culture penalizes it, extinguish it. It is this recursive relationship between individual psychological flexibility and the collective context in which it operates that the present paper places at the center of its model. The Prosocial framework provides the bridge between individual psychological flexibility and a value-based organizational culture, and it is on this bridge that the proposed model is built.

### 2.3 Organizational flexibility: state of the art and theoretical gap

The concept of organizational flexibility has a long history in the organizational behavior (OB) literature, where theorists have emphasised the need for organizations to develop structures, processes, technologies, and strategies capable of adapting across dimensions such as time, range, intention, and focus (Golden and Powell, 2000, as cited in Bond, 2018). Despite this longstanding interest, the role of employees’ psychological processes in enabling or undermining such flexibility had received comparatively little theoretical attention before Bond's (2018) contribution. Drawing on Acceptance and Commitment Therapy (ACT; Hayes et al., 1999) and Contextual Behavioral Science (CBS; Hayes et al., 2012), Bond (2018) proposed the Organizational Flexibility Model, the orgflex, as an organizational-level analogue of the individual hexaflex.

The orgflex identifies six organizational characteristics hypothesized to be functionally equivalent to the six core processes of psychological flexibility (Figure 3): purpose and goals (corresponding to values), planned action (committed action), situational responsiveness (self-as-context), effective work design (defusion), openness to discomfort (acceptance), and awareness (present-moment contact). Bond argues that organizations embodying these characteristics are better placed to pursue their purpose in a mindful, values-consistent way, and that this promotes both organizational effectiveness and employee health.

**Figure 3:**
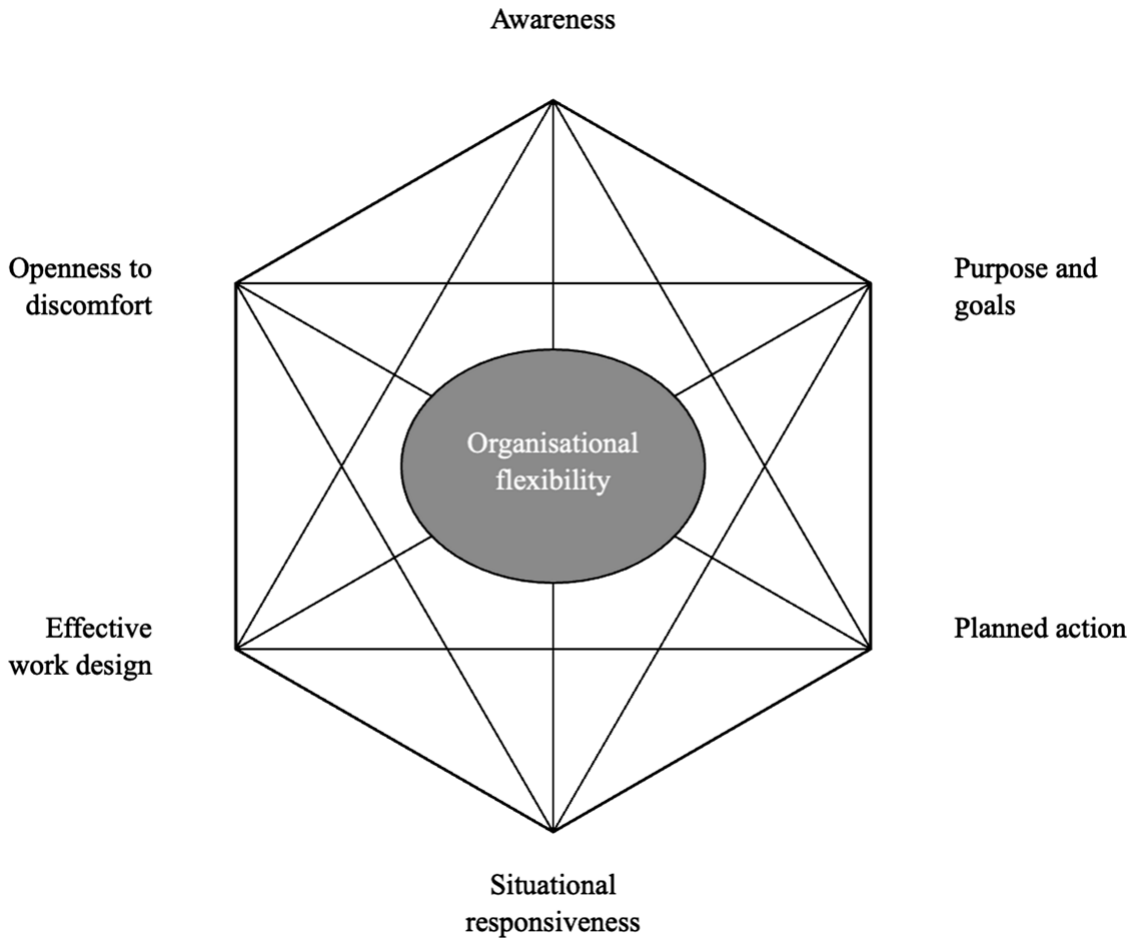
Bond’s organizational flexibility model. Conceptual model from Bond (2018).

A distinctive feature of the orgflex is its grounding in the functional-contextual commitment that theoretical constructs be operationalizable and manipulable in practice. Rather than relying on constructs such as employee motivation or the meaningfulness of work, which predict outcomes but cannot be directly influenced by practitioners, the orgflex specifies characteristics that organizations can concretely design, implement, and modify. In this respect it advances on earlier accounts of organizational flexibility, which tended to remain at a structural or strategic level without attending to the psychological dynamics that support or obstruct flexible functioning.

Bond (2018) also identifies the psychological flexibility of leaders as a condition for putting these characteristics in place and sustaining them. Psychologically inflexible leaders, he argues, are less able to tolerate the ambiguity and discomfort that genuinely flexible practices involve, such as granting employees job control or engaging openly with uncertainty. This is a cross-level link, but a top-down one: it runs from leaders to the organization they shape, and Bond notes that even this link has yet to be tested.

Bond explicitly acknowledges two limitations. First, it remains untested whether the six characteristics interact to produce organizational flexibility, or whether their combined effect on productivity and employee health operates through organizational flexibility as he proposes. Second, and of greater relevance here, the orgflex specifies how organizational characteristics may reinforce flexibility in individual workers, but not the general mechanism by which an increase in flexibility among members comes to be retained and propagated across the organization rather than absorbed and extinguished. Bond’s leader-based route is one instance of such propagation, the case in which the members who become more flexible are also those with most control over their colleagues’ conditions; what is missing is the account that would say when propagation occurs more widely and when it does not. It is this mechanism that the present paper proposes, as a hypothesis to be tested.

## 3 A multilevel theoretical model

### 3.1 Level 1

As reviewed in Section 2, psychological flexibility is a consistent predictor of occupational health outcomes. Workers with higher PF report lower burnout and greater engagement, and ACT-based interventions produce reliable improvements across occupational groups, with PF identified as the primary mechanism of change (Prudenzi et al., 2021; Towey-Swift et al., 2023). The effects of PF on occupational health outcomes depend, however, on the organizational resources available to workers. Bond and Flaxman (2006) demonstrated that PF interacted with job control in predicting learning, job performance, and mental health among call center workers: psychologically flexible workers were better able to convert available organizational resources into health and performance gains, while the same level of PF produced smaller effects in conditions of low job control. Bond et al. (2008) extended this finding in a quasi-experimental work redesign study: the intervention improved mental health and reduced absence rates, but significantly more so for workers with higher baseline PF, an effect mediated by their greater perception of job control. The implication is theoretically significant: the same individual resource produces different outcomes depending on the organizational conditions in which it is embedded. This contextual dependency raises a question that individual-level analysis alone cannot answer: what organizational conditions determine whether psychological flexibility translates into sustained health and values-based action, rather than functioning merely as a personal coping resource? It is this question that the following level of analysis addresses.

### 3.2 Level 2

The transition from individual psychological flexibility to organizational functioning requires a mechanism that links the two levels without reducing either to the other. The present paper locates this mechanism in the Prosocial framework (Atkins et al., 2019), which integrates ACT with multilevel selection theory and Ostrom’s core design principles. Within this framework, psychological flexibility does not remain a private, intra-individual resource. Through a shared method, it becomes the basis on which a group co-constructs its values. The ACT matrix (Polk, 2014) allows members to map, first individually and then together, what they care about and what they move away from, and this shared mapping is the practical route by which individually held values are extended into a common purpose. This is the work of Ostrom’s first core design principle, shared identity and common purpose, which Prosocial builds through perspective taking rather than through imposition (Atkins et al., 2019). On this account, a value-based organizational culture is not something that appears over and above its members; it is what members co-construct when a shared method turns individually held values into jointly held ones. Co-construction here does not mean reaching agreement or sharing an opinion. It means that members come to act in ways consistent with the values they have identified, and that these actions become interlocked, so that each member’s values-consistent action forms part of the conditions under which the others act. What is shared is a pattern of coordinated action, not a consensus of belief.

Central to this argument is the ACT conception of values. In ACT, values are not preferences, beliefs, or goals; they are chosen qualities of being and doing that function as ongoing directions of travel, never finally achieved but continuously instantiated through action (Hayes et al., 2006; Atkins et al., 2019). This sense of the word should be kept distinct from the way value is used in much organizational discourse, where it refers either to the economic value an organization produces or to the corporate values it lists as formal commitments. Neither of those is what is meant here. A value in the ACT sense is a direction, not an asset to be extracted and not a statement to be displayed. The distinction from goals follows from this. Goals are concrete, achievable, and exhaustible; once reached, they lose their motivational function. Values are inexhaustible: they sustain committed action across changing circumstances precisely because they cannot be possessed or completed. This difference matters for how an organization treats failure. Goals can be missed: targets are not always met and projects fail, but a value can still be pursued when a goal tied to it is missed, because the direction it specifies does not depend on any single outcome being reached. In a value-based context this keeps a missed goal available as something to learn from, one cycle in an ongoing process of experimentation, rather than an occasion to assign blame. The aversive consequence of failure is then less likely to fall on the person who missed the target and more likely to attach to whether action remains consistent with the shared direction. This reduces blaming without removing accountability: members remain answerable for acting in the service of shared values, including for correcting course after error.

Bond (2018) operationalizes this parallel at the organizational level, positioning purpose and goals in the orgflex in the same structural location that values occupies in the individual hexaflex, and arguing that organizational purpose serves the same functional role as personal values, providing a direction of travel that guides day-to-day action without ever being definitively accomplished. This functional equivalence has a direct implication. An organization whose operative direction is constituted by a genuine purpose, in the ACT sense of an inexhaustible, values-consistent orientation, will sustain committed action across uncertainty, internal conflict, and environmental pressure in ways that an organization oriented solely toward finite, measurable goals cannot. When profit is treated not as a value but as what it functionally is, a goal, its attainment does not generate further direction. Purpose, by contrast, remains generative regardless of whether proximal goals are achieved or frustrated.

A value-based organizational culture, in the sense proposed here, is one in which shared purpose works as a contextual condition that reinforces flexible, purpose-consistent action and weakens the pull toward rigid, avoidance-driven responding, at both individual and group levels. When the Prosocial method co-constructs shared values, and when the surrounding conditions reinforce rather than penalize action taken in their service, the result is a context in which purpose, not the achievement of finite targets, becomes the primary behavioral condition for organizational action. This is the Level 2 condition that the present model proposes as necessary, though not sufficient, for organizational flexibility. That flexibility is best understood not as a property the system possesses but as a pattern of coordinated, values-consistent action that is retained and propagated under conditions the next section specifies.

### 3.3 Level 3

Bond conceptualizes organizational flexibility through a set of characteristics that, as he acknowledges, have largely been theorized and studied in isolation, leaving open how they combine to produce flexibility as an integrated feature of organizational functioning. This gap is conceptual as well as empirical. A model that lists component characteristics without specifying how they cohere risks treating organizational flexibility as an aggregate, the sum of independently assessed features, rather than as a genuinely collective phenomenon. As the preceding sections have argued, individual psychological flexibility and a value-based culture are not additive inputs whose combination yields organizational flexibility by default. Organizational flexibility is not psychological flexibility scaled up, nor is it the arithmetic mean of individual flexibility scores across a workforce.

What is missing from Bond’s account is the mechanism by which individual-level processes, operating within a value-congruent culture, give rise to flexibility at the level of the organization. The concept required here is selection by consequences, applied to the coordinated action of many people rather than to the behavior of one. Organizational flexibility, on this account, is a recurring pattern of coordinated, values-consistent action that is retained and propagated when its combined product meets the conditions the wider environment selects for, and that decays when it does not. This raises an evident tension: if wider financial markets select for short-term extraction, Level 3 flexibility would seem selected against almost everywhere. Niche construction theory (Odling-Smee et al., 2003) resolves this: organizations are not passive recipients of external selection but can construct locally insulated niches, through governance structures, ownership forms, market segments answering to different metrics, or the discretionary use of leverage, that change which consequences apply to values-consistent action, without requiring the wider market to change first. This is insulation of degree, not immunity: the tension named at the outset persists, but Level 3 flexibility survives where an organization builds and defends such a boundary, and decays where it cannot. This is consistent with Morgeson and Hofmann's (1999) observation that a collective construct can share a functional equivalence with its individual-level analogue, producing similar functional outputs, while taking a structurally distinct form at the collective level. Organizational flexibility is functionally analogous to individual flexibility and structurally different from it: it is a property of how the actions of many members interlock (in behavior-analytic terms, interlocking behavioral contingencies; Glenn, 2004), not a property the organization possesses over and above them, and not a state inside any individual. Propagation, in this sense, carries a specific operational meaning: the retention over time of a values-consistent repertoire in those who acquired it, under conditions in which that repertoire carries a local cost, together with its recurrence in colleagues who were not themselves trained. What mediates both is a change in the consequences a colleague contacts, not a change in what members report.

This pattern does not arise automatically wherever flexible individuals are present. Whether an increase in flexibility is retained and spreads, or is absorbed and extinguished, depends on two conditions. The first is the position of the people in whom flexibility increases within the network of consequences: action spreads furthest when it originates with those whose role lets them alter the consequences their colleagues encounter, and travels little when it originates with those who cannot. The second is whether the surrounding culture reinforces or penalizes action taken in the service of shared values. Bond’s emphasis on the flexibility of leaders is a particular case of the first condition, the case in which the people whose flexibility increases are precisely those with the most control over their colleagues’ consequences. The present account generalizes it: leaders are the high-leverage instance of a condition that holds for any member, and the same logic explains why interventions that raise flexibility in members with little such leverage, inside a culture that penalizes values-based dissent, leave individual gains that do not propagate. Leverage does not collapse into a single vector. Vertical leverage, foregrounded in Bond’s leader-based account, is formal or informal authority over appraisals, resources, and task assignment, where consequences change fastest. Horizontal leverage is peer-to-peer accountability, formalized in Ostrom’s CDP3 (inclusive decision-making) and the graduated-sanctions principle, and shown by Wilson et al. (2013) to generalize beyond commons governance. The two are complementary: a vertically installed change is fragile without horizontal accountability to maintain it once the initiating leader moves on, while horizontal accountability alone has limited reach beyond its local unit. Hierarchy sets the ceiling and starting distribution of vertical leverage; it does not preclude, and Ostrom’s principles specifically target, the horizontal accountability operating beneath it.

Where both conditions hold, the relationship between members and culture is recursive. People who act consistently with shared values change the conditions under which their colleagues act, and those changed conditions in turn support further values-consistent action. What the social cognitive tradition describes as reciprocal determinism between persons, behavior, and environment (Bandura, 2001) corresponds, in functional terms, to operant selection operating recursively over time: each instance runs from action, to a change in the social environment, to the selection of the next action. Ostroff et al. (2012) document this co-determination of culture and behavior over time, through which repeated values-consistent action gradually stabilizes into a shared pattern, and that stability in turn lowers the local cost of further values-consistent action for individual members. The cost is lowered, not removed: acting in the service of shared values continues to carry immediate local costs, the discomfort of dissent or the short-term gain foregone, but a culture that reinforces such action at the aggregate level shifts the net consequence so that those local costs no longer dominate.

This is where a value-based culture differs from one that merely secures compliance, and the difference is not the presence or absence of rules. Every organization, value-based or not, runs on a mixture of real consequences that members deliver to one another, verbal rules that specify what to do, and shared statements of purpose that change how much those consequences matter. What distinguishes the two kinds of culture is the composition and function of this mixture. A culture that governs mainly through rules backed by surveillance produces action that holds while members are watched and the threat is credible, and lapses when either fails. A culture in which shared purpose genuinely changes what reinforces members produces action in the shared direction that holds even when no one is watching, because what sustains it is not fear of whoever issued the rule. The two can look identical from outside, members doing things aligned with a stated purpose. They separate when surveillance drops: compliance fades, values-consistent action persists. This observable difference is what the present model means by a value-based culture, and it is one a purely extractive culture cannot produce, because surveillance-backed rules are the only instrument it has.

This gives the third level a resemblance to the first that is worth stating carefully, because the resemblance is functional and not a matter of the same operation. At the individual level, values-consistent action persists in the presence of difficult private experiences because a context, supplied by defusion and acceptance, alters the function those experiences have over behavior while they remain present. At the organizational level, values-consistent action persists in the presence of adverse local consequences because a context, supplied by the shared culture, alters the function those consequences have over behavior while they too remain present. The shared form is that a wider context changes the function of an adverse event that is still there, so that it no longer dominates action. The mechanisms differ: in one case the context bears on private events within a person, in the other on consequences exchanged between people. The parallel is offered as a way of seeing the continuity across levels, not as a claim that the same process operates at each.

The outcomes of this alignment are observed at the level of the group as a whole and are not recovered by summing individual wellbeing scores. Danna and Griffin (1999) distinguish organizational health from individual wellbeing: individual wellbeing refers to the states experienced by members, whereas organizational health refers to how the organization goes on functioning over time, adapting to changing demands and maintaining the conditions that support its members. Read functionally, this names not a property the organization holds but a description of coordinated action that continues: purpose-consistent action that persists under environmental pressure, adaptation that preserves rather than abandons shared purpose, and coordination that survives disruption. These outcomes feed back into the conditions that produced them, in the recursive way described above. Specifying how they can be measured, at the right level and independently of the alignment said to produce them, is the principal task the model leaves to future research, and it is taken up in the propositions that follow.

## 4 Theoretical propositions

*Proposition 1: Individual psychological flexibility is positively associated with worker health.*

This proposition states the established premise on which the rest of the model builds; it is not a contribution of the paper. As Sections 2.1 and 3.1 set out, psychological flexibility correlates with better occupational health outcomes and ACT-based interventions reliably improve it. In functional terms, flexibility supports health because it enables values-consistent action and weakens experiential avoidance under workplace stressors. Worker health here refers to individual-level outcomes such as burnout, stress, and wellbeing, kept distinct from the organizational-level health addressed by Proposition 5. The role of this proposition is structural rather than informative: without an established individual effect, the propagation account that follows would have nothing to propagate.

*Proposition 2: Applying the Prosocial method extends individually held values into jointly held ones within the group.*

This is the one proposition in which the paper claims an effect of a specific intervention, and it forms the bridge from the individual to the collective level. The method combines the ACT matrix with Ostrom’s core design principles, and its effect is measured through shared identity and common purpose, named after Ostrom’s first core design principle. Jointly held values, in the sense used here, does not mean agreement or shared opinion; it means that members come to act in ways consistent with the values they have identified and that these actions become interlocked. Shared identity and common purpose, named after Ostrom’s first core design principle, are therefore measured not as a collective state of mind but through their behavioral signature: a self-report index treated as verbal behavior, and a behavioral convergence index capturing the degree to which members’ actions move toward common products even at local cost. To avoid circularity, the target repertoires against which convergence is scored are identified, preliminarily, from a source independent of the trained group, so that the intervention does not define its own criterion of success. Testing the proposition requires a comparison condition: applying the method to flexible individuals should increase convergence relative to a control. This convergence index is also a first step toward the systemic outcome measure of Proposition 5, but it is operationally distinct, indexing here the group-level adoption of shared values rather than sustained organizational functioning.

*Proposition 3: A value-based organizational culture moderates the relationship between individual psychological flexibility and worker health, strengthening it: where the culture reinforces values-consistent action despite its local costs, individual flexibility translates into health more fully than where the culture penalizes such action. What does the moderating work is the value content of the culture, not the sheer quantity of resources or job control available.*

This is the first of the model’s two substantive contributions: it specifies the condition under which the individual effect of Proposition 1 is realized or suppressed. Culture here has the contingency-structure definition it shares with Proposition 4: the consequences members deliver to one another, together with the rules and shared statements of purpose that modulate their function. A value-based culture, in these terms, is one that reinforces values-consistent action despite its local cost, not one that merely declares values. The moderation works not by amplifying an effect already present but by shifting the sign of the net contingency: where the culture offsets the local cost of values-consistent action through aggregate reinforcement, individual flexibility can translate into sustained action and so into health; where the culture penalizes that action, the same flexibility yields less. The cost is contacted, not removed. The final clause is the proposition’s differential prohibition and is what makes it falsifiable: what moderates is the value content of the culture, not the quantity of resources or job control, placing it in direct competition with accounts in which job control does the moderating work (Bond and Flaxman, 2006). It is falsified if, in a high-control but low-value-content setting, flexibility translates into health as fully as in a value-based one. This proposition concerns individual health only; how values-consistent action spreads beyond the individual worker is the separate question taken up by Proposition 4.

*Proposition 4: Whether an increase in individual psychological flexibility is retained and spreads across the organization, rather than being absorbed and extinguished, depends jointly on two conditions: the leverage of the affected members over the consequences experienced by others, and whether the surrounding culture reinforces or penalizes values-consistent action.*

This is the central theoretical contribution of the paper, the propagation mechanism offered as a hypothesis, and the bridge between individual flexibility and organizational functioning. Leverage is greatest for those whose role lets them set the reinforcement and punishment co-workers encounter. Where both conditions hold, values-consistent action is sustained and propagates; where flexibility is raised in low-leverage members inside a culture that penalizes values-based dissent, the gain stays individual and decays. Leverage means effective control over the consequences others encounter, not position on the organizational chart: an informal member on whom others’ outcomes depend has leverage, a nominal manager stripped of real control does not. This is what makes Bond’s leader-based route a special case rather than the rule. The culture term carries the same contingency-structure definition as in Proposition 3. The mechanism asserted is not a change in colleagues’ attitudes but a change in the contingencies a high-leverage node arranges: whether, having become more flexible, that member actually alters the reinforcement and punishment colleagues encounter; this disposed change in consequences, not reported attitudes, is the mediator to be measured. The proposition is falsified if raising individual flexibility in members with little control over their colleagues’ consequences, inside a culture that penalizes values-based dissent, nonetheless produces values-consistent action that is retained over time and taken up by others rather than decaying. The test is anchored to two observables, whether the repertoire is retained rather than extinguished in those trained, and whether it appears in colleagues who were not, and to no systemic organizational outcome, which belongs to Proposition 5. Leverage must be measured *a priori* and independently of the outcome, through an index of who controls whose consequences: who sets appraisals, who allocates resources, who grants or withholds what colleagues work for. Without this independent measure the proposition becomes circular, confirmed after the fact by the very spread it is meant to predict. This is the most demanding part of the design, and is declared as such.

*Proposition 5: The organizational flexibility sustained by this alignment is associated with forms of functioning observed at the level of the group as a whole, not recovered by summing individual wellbeing scores: purpose-consistent action that persists under environmental pressure, adaptation that preserves rather than abandons shared purpose, and coordination that survives disruption. Each can serve as a test of the model only if its threshold is fixed in advance and measured independently of the alignment from which it is said to follow. Developing such measures is the primary methodological task the model sets for future research.*

Unlike the others, this proposition is not yet a test-ready prediction, and the paper says so plainly: its first task is not to be tested but to make testable the outcome it names. Until a measure of systemic functioning exists that is independent of the alignment said to produce it, Proposition 5 is a direction for research rather than a falsifiable hypothesis. Stating this does not weaken it; it distinguishes the model from accounts that posit systemic outcomes and then measure them with individual-level proxies. The functioning at issue is described throughout as coordinated action that continues, not as a capacity the organization possesses: purpose-consistent action that persists under pressure, adaptation that preserves rather than abandons shared purpose, and coordination that survives disruption. The primary task the proposition sets is therefore to build the measure, at the level of the group and independently of the alignment from which the outcome is said to follow; measuring systemic functioning through that alignment would yield a tautology rather than a test. This independence must also hold between Propositions 4 and 5: the propagation of Proposition 4 is tested through retention and uptake of the repertoire, the outcome of Proposition 5 through systemic functioning, and neither may be measured through the other, on pain of making the model as a whole unfalsifiable. The empirical program this implies, including the factorial separation of leverage and culture and the development of the systemic measure, is set out in the next section.

## 5 Implications

### 5.1 Implications for future research

The model developed here is a set of hypotheses, and this section sets out what testing them would require. Because its central claim, Proposition 4, makes the fate of an increase in individual flexibility depend jointly on two conditions, the leverage of the affected members over the consequences others encounter and whether the surrounding culture selects for or against values-consistent action, the natural design is factorial: leverage, high or low, crossed with culture, value-based or extractive. The purpose of such a design is not only to test whether propagation occurs, but to separate the two conditions from one another and to set the joint model against the single-factor accounts with which it would otherwise be confused.

Before any of this can be carried out, leverage has to be measured, and measured in a way that does not presuppose the outcome. This is the precondition on which the whole program rests, and it is the most fragile point in the design. Leverage, as defined here, is effective control over the consequences a person’s colleagues encounter: who sets their appraisals, who allocates the resources they depend on, who can grant or withhold what they work for. An adequate measure would map these relations of consequence-control directly, for instance through network methods that trace who depends on whom, rather than reading leverage off the organizational chart, which records authority but not the actual distribution of consequences. Developing and validating such a measure, prior to and independently of any propagation it is later used to predict, is the first methodological task the program sets itself; without it, the factorial design rests on an unoperationalized construct, and any apparent confirmation would be circular.

Within the factorial frame, three studies would do the work. The first is a negative control: individual-focused training delivered to low-leverage members in a culture that penalizes values-based action; the model predicts the resulting action stays with the trained individuals and decays, and is falsified if it instead spreads. The second isolates leverage: culture held constant and value-based, leverage varied; the model predicts propagation tracks leverage, and is falsified if propagation is equal regardless of leverage. The third isolates culture: leverage held high, the cultural frame varied while training is held constant; the model predicts propagation tracks the cultural frame, and is falsified if propagation occurs equally regardless of it.

These three studies test the propagation of a repertoire, through its retention in those trained and its uptake by those not trained, which is the territory of Proposition 4. The full test of Proposition 5 remains suspended until its own precondition is met: a measure of systemic functioning, defined at the level of the group and independent of the alignment said to produce it. Building that measure is a task in its own right, and until it exists the systemic outcome can be described and motivated but not tested. Keeping the two apart, propagation measured through retention and uptake, systemic functioning through a yet-to-be-developed group-level index, is what allows Propositions 4 and 5 to be tested separately rather than each through the other, and is what keeps the model as a whole open to disconfirmation.

### 5.2 Implications for practice

If the model is right, the most consequential implication for practice is also the least convenient: raising the psychological flexibility of individuals, however well done, is not on its own a reliable route to organizational change. What decides whether such gains take hold is where they land and what selects them. Two priorities follow. The first is to identify the members whose roles give them real control over the consequences their colleagues encounter and to treat their flexibility as the high-leverage point it is, rather than spreading intervention evenly across a workforce on the assumption that individual change aggregates. The second is to work on the contingencies themselves: an intervention that leaves untouched the consequences a culture attaches to values-consistent action will find that those consequences, not the intervention, decide the outcome. The Prosocial method discussed earlier is one concrete vehicle for this second task, in that it works directly on shared purpose and on the norms governing how members treat one another’s values-consistent action, rather than on individuals in isolation.

This places a particular demand on those at the top of the leverage structure. Because leadership typically holds the greatest control over the consequences others encounter, a culture that selects for values-consistent action cannot be built on leadership’s indifference to it: the value choice has to be made there first. Concretely, this means treating worker health as a value rather than as an instrument, as something the organization is committed to in its own right and not only when, and for as long as, a given worker is profitable. This is a strong commitment, and the model does not soften it: to make health conditional on the profit a worker returns is, in the terms used here, to treat health as a goal, exhaustible and contingent on an outcome, rather than as a value, a direction pursued regardless of whether any particular outcome is reached. The point is not that an organization abandons profit or ceases to act on it, but that health is not subordinated to it; an organization can hold both, profit as a goal and health as a value, just as this paper has distinguished the two throughout. A culture in which leadership protects health only insofar as it pays is, on this account, not a value-based culture at all, however it describes itself, and the diagnostic of behavior under reduced oversight would expose it as such.

The model also gives practitioners a way to tell whether a culture genuinely supports values-consistent action or merely secures compliance with it, and the test is observable rather than declarative. A culture that produces compliance keeps behavior in line while members are watched and the relevant pressure is credible; a culture in which shared purpose has genuinely changed what reinforces members keeps it in place even when no one is watching. The diagnostic is therefore what happens when oversight relaxes: where aligned behavior fades, the organization has been buying compliance; where it persists, the underlying contingencies have shifted. Mission statements and declared values reveal little here; behavior under reduced oversight reveals a great deal.

Two cautions attach to these implications. The first concerns their rationale. The case for attending to organizational health made here does not rest on a demonstrated return to performance or profit, and practitioners drawn to the model should resist re-importing that justification through the back door. The position taken throughout is that worker health is worth pursuing in its own right, and the practical value of the model does not depend on its eventually paying for itself. The second concerns the use of leverage. That change propagates through high-leverage members is not a license to engineer compliance more efficiently through them. The object the model holds up is values-consistent action that persists without surveillance, which is exactly what cannot be manufactured by tightening contingencies around people. Using the leverage of key members to build genuine value-based contingencies, and using it to enforce conformity, are different undertakings, and only the first is what the model recommends.

### 5.3 Limitations

This paper is theoretical. It assembles established processes into a new mechanism and derives hypotheses from it, and it is the mechanism, not the processes it draws on, that has yet to be tested. The central claims, the propagation mechanism of Proposition 4 and the systemic outcome of Proposition 5, are offered as hypotheses, and the value of the model, if it has any, lies in making them precise enough to be wrong rather than in establishing that they are right.

The processes the model builds on are not unevidenced. Psychological flexibility and its measurement, the ACT matrix as a method, and the multilevel-selection framework on which the Prosocial approach rests are all established, and selection by consequences is foundational to behavior analysis and integral to process-based accounts of ACT rather than novel to this paper. What has seldom been measured is propagation itself: whether, and through what conditions, a change produced in some members comes to be retained and taken up across an organization. The motivating observation, that individual-focused interventions improve individual flexibility without reliably changing organizations, therefore rests not on an absence of evidence about the component processes but on the thinness of evidence about this single link. The model treats propagation as an open question, and evidence of it occurring in ways the model does not anticipate would count against it.

The program the model implies depends on two measures that do not yet exist. Leverage must be assessed in advance and independently of the propagation it is used to predict, and systemic functioning must be assessed at the level of the group and independently of the alignment said to produce it. Both are difficult, and until they are built and validated the central claims cannot be put to a clean test. This is not a peripheral caveat: the two hardest constructs in the model are also the two on which its testability depends.

Several further limitations qualify the argument. The parallel drawn between the individual and organizational levels is one of functional equivalence with structurally distinct mechanisms, not of identity; it is offered to organize understanding, no proposition rests on it, and it should not be read as a claim that the same process operates at each level. The treatment of culture as a structure of contingencies, combining the consequences members deliver to one another, the rules they follow, and the shared statements of purpose that modulate them, is a deliberate simplification of a heterogeneous reality, and the clean separation of value content from resources that Proposition 3 requires may prove difficult to achieve in practice. Several of the frameworks the model draws together are already connected in the literature, the Prosocial approach itself joining ACT with multilevel selection; what is new is the propagation mechanism assembled from them and its application to organizational flexibility, and the cross-level functional equivalence on which it leans is assumed rather than demonstrated. Finally, the individual-level association on which Proposition 1 builds is drawn largely from cross-sectional evidence, and the directional language used elsewhere in the model should be read with that base in mind.

## 6 Conclusion

Individual psychological flexibility improves the health of the person who has it, but it does not, on its own, reliably change the organization that person belongs to. This paper has taken that disparity not as a failure of individual intervention but as a question its proponents have rarely posed: under what conditions does a change produced in some members come to be retained and taken up across an organization, rather than absorbed and extinguished? The answer it proposes is that propagation, not production, is what bridges individual flexibility and organizational functioning, and that propagation depends on two conditions, the leverage of the members in whom flexibility increases over the consequences their colleagues encounter, and whether the surrounding culture selects for or against values-consistent action.

The model assembles established components, psychological flexibility, the Prosocial method, and selection by consequences, into a single account in which an organization does not possess flexibility as a property but sustains it as a pattern of coordinated, values-consistent action that the wider context either retains or lets decay. Read this way, Bond’s leader-based route becomes a high-leverage special case of a more general mechanism, and a value-based culture is distinguished from one that merely secures compliance not by what it declares but by what its members do when no one is watching. The stance the paper takes is explicit: worker health is treated as a value worth pursuing in its own right, not as an instrument whose protection is contingent on the profit it returns.

What the paper offers is a set of hypotheses, not a set of findings. The propositions are framed to be testable, and the two measures on which a clean test depends, an *a priori* index of leverage and a group-level index of systemic functioning, are named as the first tasks any such test must complete. The contribution is to specify, precisely enough to be wrong, how individual flexibility might come to shape an organization; whether it does is a question the model is built to make answerable.
